# Use of Kefir in Cheese Ball Production: Physicochemical, Textural, Rheological, and Sensory Properties

**DOI:** 10.1002/fsn3.70183

**Published:** 2025-04-10

**Authors:** Hilal Çavuş, Ahmet Emirmustafaoğlu

**Affiliations:** ^1^ Department of Gastronomy and Culinary Arts Bolu Abant Izzet Baysal University Bolu Turkey

**Keywords:** cheese ball, concentrated kefir, edible kefir, rheology, texture, white cheese

## Abstract

In this study, kefir was transformed into edible cheese balls with improved physicochemical, sensory, nutritional, and functional properties in hopes of increasing consumer diversity. For this purpose, kefir was concentrated with a solid–liquid centrifuge machine. We produced the cheese balls by mixing concentrated kefir and white cheese in the ratios of 0:100 (K0), 25:75 (K25), 50:50 (K50), 75:25 (K75), and 100:0 (K100) and characterized them in terms of their physicochemical, textural, rheological, and sensory properties. We found that the group with the highest values in terms of dry matter, protein, energy, pH, b*, chroma (C*), hardness, and gumminess values was K100, followed by K75, and the lowest values, except for the hardness value, were K0. The results showed that the cheese ball samples exhibited viscoelastic solid and non‐Newtonian character, and K100 and K25 had the highest storage modulus (*G*′) and complex viscosity (η*) values. K75 sample was found to have the lowest *G*′ and viscous modulus (*G*″) values. We found that K100 demonstrated the highest scores in terms of color‐appearance (4.51), structure‐consistency (4.23) and general acceptability (4.18) criteria on a 5‐point hedonic scale (1 = very bad and 5 = very good). K75 (4.15) was the most liked group in the taste‐odor criteria, followed by K100 (4.13). The use of concentrated kefir in cheese ball production increased the dry matter, protein, carbohydrate, energy, pH, gumminess values, and all sensory evaluation scores of the samples. The study concluded that concentrated kefir can be used alone or mixed with white cheese in different proportions in cheese ball production, resulting in the nutritional value and sensory properties of the product increase.

## Graphical abstract

In this study, we produced cheese balls by mixing kefir with white cheese in different ratios (0%–25%–50%–75%–100%) to improve the consumption diversity of kefir, and the physicochemical, textural, rheological, and sensory properties of cheese ball samples were examined. We determined that the protein, carbohydrate, energy, and sensory appreciation values increased, and the salt content decreased as a result of the use of concentrated kefir in cheese ball production. Concentrated kefir can be used alone or mixed with white cheese in different proportions to obtain cheese balls with high sensory, nutritional, and functional properties.

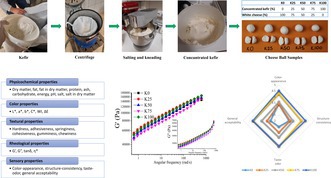

## 1 Introduction

Cheese ball is a healthy snack made by mixing different cheeses and some spices and herbs. It is usually made from white cheese. Spices and herbs can change according to the desired taste and aroma and may include sesame, pepper, chives, black cumin, and parsley. It can be consumed alone as well as with ingredients, such as eggs and flour by baking or frying. Due to these materials in its composition, cheese ball with a rich taste and aroma can be used in cold buffets domestically and in eatery outlets. At the same time, cheese balls are obtained to evaluate the excess cheese in the businesses and are offered for the consumption of the customers (Turan and Türkay 2024). It is a product liked by both adults and children and is a good source of protein, vitamins, and minerals.

Kefir is a dairy product with a unique taste and aroma, obtained as a result of lactic acid and ethyl alcohol fermentations. The origin of kefir is considered to be the Balkans, Eastern Europe, and the Caucasus. Due to the positive properties of kefir on health, its consumption has spread to other parts of the world (Viogenta et al. 2021). Kefir is thought to be more advantageous to consume compared with other fermented dairy products, such as yogurt or cheese, due to the health‐beneficial bioactive compounds and complex and specific microorganisms it contains (Mitra and Ghosh 2021).

It is known that kefir, being a functional drink, contains a balanced content of protein, fat, and carbohydrates, and is also rich in vitamins and minerals. Researchers have found that kefir consumption can offer benefits of antimicrobial (Lakshmi et al. 2017; Azizi et al. 2021), antitumor (Rajoka et al. 2019), anticarcinogenic (García‐Burgos et al. 2020), hypocholesterolemic (Yegin et al. 2022), antidiabetic (Farag et al. 2020; Salari et al. 2021), and immunomodulatory activity, as well as improving lactose digestion. Despite all this, consumers should understand that functional foods are not a quick fix or a panacea for poor health habits (Abedinia et al. 2025).

Recently, people have tried to include more functional foods that have high nutritional value, are beneficial to health, and strengthen the immune system in their diets (Vieira et al. 2021; Ganatsios et al. 2021). As a result, fermented dairy products like kefir have gained popularity. Consumption of kefir can be limited for some consumers because of situations, such as their dislike of its taste and difficulty in drinking it. Concentrating kefir and producing it in different forms can lead to an increase in its consumption. However, studies with this focus are quite limited. Aktaş et al. (2024) produced concentrated spreadable kefir from cow, goat, sheep, and buffalo milk in a cloth bag and examined the microbiological, physicochemical, and sensory properties of the samples. Methods, such as evaporation, membrane filtration, filtering in a cloth bag, adding milk powder, and centrifugation, are used to concentrate dairy products (Tamime et al. 2007). The main purpose of the production of concentrated dairy products is to remove the serum phase contained in the product and obtain a product with high dry matter. Operations leading to the increase in dry matter proportion cause the protein and fat content to increase as the water, lactose, and some mineral substances in real solution form in the milk are separated from the product (Ozer, 2006).

The aim of this study was to use concentrated kefir to improve the cheese ball's physicochemical, sensory, nutritional, and functional properties. Thus, kefir will offer an alternative consumption other than a drink. The physicochemical, textural, rheological, and sensory properties of cheese balls produced by combining concentrated kefir and white cheese in various ratios (0:100, 25:75, 50:50, 75:25, and 100:0) were investigated.

## 2 Material and Methods

### 2.1 Material

Three percent fat UHT cow milk (İçim, Turkiye), full‐fat white cheese (Sütaş, Turkey), and kefir culture (Vivo, Ukraine) used in kefir production were all purchased from the market. Kefir culture consists of *Lactococcus lactis ssp. lactis, Lactococcus lactis ssp. cremoris, Lactococcus lactis ssp. Lactis var. diacetylactis, Leuconostoc mesenteroides ssp. cremoris, Streptococcus thermophilus, Kluyveromyces marxianus ssp. marxianus*, and *Debaryomyces hansenii* .

Isoamyl alcohol (CAS 123‐51‐3; Merck) and Gerber centrifuge (Gerber Instruments, Micro II, Switzerland) were used for fat determination; sulfuric acid (95%–97%, CAS 7664‐93‐9; Merck) was used for fat and protein determination. In protein determination, the combustion unit (DK‐20; Velp Scientifica), distillation unit (UDK‐139; Velp Scientifica), hydrochloric acid (37%, CAS 7647‐01‐0; Sigma‐Aldrich), boric acid (CAS 10043‐35‐3), Kjeldahl catalysis agent (A00000274; Velp Scientifica), methyl red (CAS 493‐52‐7; Merck) and bromocresol green (CAS 76‐60‐8; Merck) were used. Silver nitrate (CAS 7761‐88‐8; Merck), and potassium chromate (CAS 7789‐00‐6; Merck) were used for salt determination.

### 2.2 Kefir Production and Concentration

The milk was heated until it reached 27°C, and 0.54 g/L kefir culture was added. It was then incubated at 25°C. When the pH reached 4.5 after 16–17 h, the kefir samples were removed from the incubator and kept at +4°C in a domestic refrigerator for 48 h. Refrigerated kefirs were poured into cotton bags (standard cotton American cloth). Centrifugation was performed at 200 *g* for 1 h, then at 400 g for 1 h, and finally at 600 g for 1 h in the solid–liquid centrifuge machine (Emirmustafaoğlu and Coşkun 2017).

### 2.3 Cheese Balls Production

The concentrated kefir was taken into the kneading machine with the addition of 1% salt and mixed for 1 min. Preliminary tests were conducted to determine the optimum salt content, and the ideal salt content was determined as 1%. The cheese was taken into a food processor and mixed with the chopping apparatus for 1 min. Cheese ball samples were prepared in two replicates by mixing white cheese and concentrated kefir in the proportions specified in Table 1. Cheese ball samples were stored in lidded plastic containers at +4°C until analysis (< 3 days). No packaging was applied to the final product.

**TABLE 1:** Cheese ball samples.

Groups	Concentrated kefir (%)	White cheese (%)
K0	0	100
K25	25	75
K50	50	50
K75	75	25
K100	100	0

### 2.4 Physicochemical Characterization

Dry matter and ash content were determined by the gravimetric method at 105°C and 550°C, respectively. Fat determination was made by the Gerber method, and protein determination was made by the Kjeldahl method (Metin and Öztürk 2017).

The amount of carbohydrates was found by subtracting the sum of the amounts of fat, protein, and ash from the amount of dry matter. The energy value was determined by applying the following formula:

$$\text{Energy}\;\left(\text{kcal}\right)=\text{Carbohydrate}\%\times 4+\text{Protein}\%x\;4+\mathrm{Fat}\%\times 9$$

A pH meter (Milwaukee, MW102, Romania) was used for pH determination. Salt determination was made by the Mohr method (Metin and Öztürk 2017).

### 2.5 Color Analysis

L* (brightness), a* (redness or greenness) and b* (yellowness or blueness) values of the samples were measured using a color analyzer (CR400; Konica Minolta, Japan). Whiteness index (WI) and Chroma (C*) values derived from these parameters as (Aydemir and Kurt 2020):

$$\mathrm{WI}=100-\sqrt{{\left(100-L^{*}\right)}^{2}+{\left(a^{*}\right)}^{2}+{\left(b^{*}\right)}^{2}}$$

$$C^{*}=\sqrt{{\left(a^{*}\right)}^{2}+{\left(b^{*}\right)}^{2}}$$

To reveal the color difference between samples, Δ*E* value was calculated for cheese ball samples using the following equation (Chudy et al. 2019). The Δ*L*, Δ*a*, and Δ*b* values were calculated by taking into account the L*, a* and b* values measured for the trial samples and taking K0 as the control sample.

$$\Delta E=\sqrt{∆L^{2}+∆a^{2}+∆b^{2}}$$

### 2.6 Texture Analysis

A texture analyzer (Stable Microsystems, Surrey, UK) was used to determine the textural parameters of the samples. The Texture Profile Analysis (TPA) method was used. In measuring the hardness, adhesiveness, springiness, cohesiveness, gumminess, and chewiness values of the samples, the method specified by García‐Gómez et al. (2020) was modified. The cheese ball samples were shaped into spheres with a diameter of 25 mm, and measurements were made at 15°C. Analyses were carried out using a 2 kg load cell and P36R cylinder probe, and 50% compression was applied. The pretest speed was 1 mm/s, the test speed was 1 mm/s, the final test speed was 2 mm/s, the test duration was 5 s, and the trigger force was 5 g.

### 2.7 Rheological Analysis

The rheological properties of the samples were determined using the Anton Paar Rheometer (Physica, MCR 302; Anton Paar GmbH, Austria) by modifying the method reported by Mazinani et al. (2021). Strain sweep and frequency sweep tests were performed using 25 mm diameter parallel plate geometry at 4°C with a gap size of 1 mm. A small slice of cheese ball was placed on the bottom plate; then, the top plate was slowly moved downward. Excess cheese was carefully removed with a scraper and the sample was left to rest in the rheometer for 5 min. To find the linear viscoelastic range, dynamic sweep strain testing was performed at 4°C with a strain range of 0.01%–10% and a frequency of 0.1 Hz. Then, a screening test was performed in the angular frequency (ω) range of 0.628–628 rad/s by selecting a strain in the linear region (0.2%) and the storage modulus (*G*′), viscous modulus (*G*″), tan (*δ*), and complex viscosity (*η⃰*) values were recorded. The data obtained were modeled against angular frequency with the following power law model.

$$G^{\prime }=K^{\prime }{\mathrm{\omega n}}^{\prime }$$

$$G^{\prime \prime }=K^{\prime \prime }{\mathrm{\omega n}}^{\prime \prime }$$

$$\eta^{*}=K^{*}\omega n^{*-1}$$

### 2.8 Sensory Analysis

A scoring test was performed on the cheese ball samples in terms of color‐appearance, structure‐texture, taste‐odor, and general acceptability. Sensory analyses were carried out with a semi‐educated panelist group of 40 people, 19 men and 21 women, aged between 18 and 59. Panelists made their evaluations by giving points between 1 and 5 (1 = very bad and 5 = very good) (Stone et al. 2020). The study was approved by the Bolu Abant İzzet Baysal University Ethics Committee with registration number: 2022/41.

### 2.9 Statistical Analysis

The data obtained from the measurements above were subjected to the analysis of variance (ANOVA) and then to the Tukey post hoc test at the *p* < 0.05 level.

## 3 Results and Discussion

### 3.1 Physicochemical Properties

The physicochemical analysis of produced cheese balls is presented in Table 2. K0 has the lowest dry matter value (32.38%) and K100 has the highest (35.10%). The dry matter value has been shown to increase with a higher concentration of kefir and decrease with an increasing content of white cheese. The higher protein content of K100 increased its water holding capacity and led to an increase in dry matter value. Water holding capacity is the ability of proteins and other hydrocolloids to retain free water without exudation or syneresis (Amador‐Espejo et al. 2021). In determining the filtration parameters to be applied to kefir, it was aimed to reach a dry matter value close to white cheese. Centrifuge parameters can be changed to obtain higher or lower dry matter values for centrifuged kefir. The dry matter value of white cheese was found to be between 30.28% and 36.23% by Nasiri et al. (2020) and between 34.16% and 59.63% by Çetinkaya (2021). The dry matter value of white cheese is influenced by the production process and the dry matter ratio of the milk used. Şanlı and Anlı (2020) found the dry matter value of Çökelek cheese, which they produced using kefir, to be between 23.97% and 24.54%. In this study, the separation of kefir fat and the separation of a small amount of water with a 12h filtration process led to lower dry matter results than our study. Öztürkoğlu‐Budak et al. (2021) added rennet to kefir to coagulate it, then filtered it for 16–22 h to produce Quark. They found the dry matter values of the Quark produced in this way to be between 21.2% and 24.6%. The centrifugation method specified by Emirmustafaoğlu and Coşkun (2017) and applied in our study enabled the dry matter value to be reached around 34% in a short time of 3 h.

**TABLE 2:** Physicochemical properties of cheese balls.

	Cheese ball samples ($\bar{X}$ + SD)				
	K0	K25	K50	K75	K100
Dry matter (%)	32.38 ± 0.364^a^	32.63 ± 0.175^a^	34.11 ± 0.331^b^	34.41 ± 0.962^ab^	35.10 ± 1.838^ab^
Fat (%)	15.25 ± 0.5^a^	15.25 ± 0.289^a^	15.50 ± 0.408^a^	15.00 ± 0.408^a^	15.00 ± 0.913^a^
Fat in dry matter (%)	47.11 ± 1.623^c^	46.73 ± 0.9^c^	45.44 ± 1.057^bc^	43.61 ± 1.118^ab^	42.74 ± 0.935^a^
Protein (%)	11.04 ± 0.145^a^	11.37 ± 0.367^a^	12.72 ± 0.609^a^	13.38 ± 1.212^a^	14.39 ± 1.615^a^
Ash (%)	2.83 ± 0.017^e^	2.48 ± 0.05^d^	2.06 ± 0.022^c^	1.79 ± 0.028^b^	1.48 ± 0.026^a^
Carbohydrate (%)	3.27 ± 0.731^a^	3.54 ± 0.274^a^	3.83 ± 0.401^a^	4.24 ± 0.473^a^	4.23 ± 0.752^a^
Energy (kcal)	194.44 ± 2.958^a^	196.87 ± 1.766^a^	205.70 ± 2.935^b^	205.47 ± 5.201^ab^	209.45 ± 11.746^ab^
pH	4.40 ± 0.02^a^	4.45 ± 0.012^b^	4.47 ± 0.012^bc^	4.50 ± 0.021^c^	4.50 ± 0.019^c^
Salt (%)	2.08 ± 0^d^	1.85 ± 0^c^	1.56 ± 0.115^bc^	1.21 ± 0.12^ab^	0.86 ± 0.115^a^
Salt in dry matter (%)	6.42 ± 0.071^e^	5.66 ± 0.03^d^	4.57 ± 0.306^c^	3.52 ± 0.284^b^	2.47 ± 0.293^a^

*Note:* Means shown with different letters in the same row are statistically different (*p* < 0.05).

Abbreviations: $\bar{X}$: mean; SD, standard deviation.

The fat values of the cheese ball samples were between 15.00% and 15.25% (Table 2). There is no statistically significant difference between the samples' fat values (*p* > 0.05). In recent years, in line with people's tendency to reduce their saturated fat intake, studies have been carried out on alternatives, such as vegetable oil, oleogel that can replace saturated fats in dairy products (Qi et al., 2021). Sunflower seed oil is widely appreciated in many countries in Europe, Mexico, and South America compared with other vegetable oils due to its easy availability and various health benefits, such as lower serum cholesterol, low‐density lipoprotein levels, antioxidants, blood pressure regulation, anti‐inflammatory effects, skin protectant, and pain relief (Li et al. 2024). Unsaturated fatty acids, which are found in large quantities in vegetable oils, are prone to forming trans isomers during processing and storage. Trans isomers are harmful to the human body, and many countries have limited their amount in foods (Li et al. 2023). The type of fat affects the sensory, textural, and rheological properties of cheese. Fat in cheese is an important parameter affecting quality characteristics, such as taste, texture, melting properties, and general appearance (Lorenzen et al. 2025). The fat content of the white cheese, the fat content of the milk, and the centrifuge parameters affect the fat content of the cheese ball. In our study, concentrated kefir produced from 3% fat milk produced the cheese ball. Different researchers reported the fat value of white cheese as 15.00%–15.50% (Gümüş and Hayaloğlu 2019), 15.01%–21.00% (Çetinkaya 2021). White cheese with different fat content is produced by varying the fat content of the milk used in production.

K0 had higher fat in dry matter value than other samples (*p* < 0.05). As the concentrated kefir ratio in the cheese ball composition increased, the fat in dry matter values decreased. The fat in dry matter content of cheeses greatly affects their consistency (Černíková et al. 2017). As the concentrated kefir ratio used increased, the dry matter ratio increased, while the fat ratio remained almost unchanged, causing the fat ratio in the dry matter to decrease from K0 to K100. The current fat in dry matter values agree with the findings on white cheese in the literature (Aydemir 2018; Çetinkaya 2021).

The protein values of the cheese balls produced using concentrated kefir were found to be higher than the control group produced using white cheese (Table 2). The protein content of cheese balls increased in line with the usage of kefir. The protein value, which was 11.04% in the control group, increased to 14.39% in K100. The results obtained are parallel to the dry matter values. The high rate of water separation during kefir concentration increased the dry matter, and as a result, the protein value increased as the rate of concentrated kefir was increased. Fermentation of milk with kefir culture leads to an increase in protein content due to the release of protein fragments resulting from the activity of both kefir microorganisms and natural milk proteases. The protein content of cow milk increased by 38.5% after 24 h of fermentation with kefir culture (La Torre et al. 2024). Accordingly, the protein content of the samples using concentrated kefir was found to be higher in our study. Demir and Özkısa (2020) reported protein content ranging from 9.04% to 10.86% for filtered kefirs, whereas Öztürkoğlu‐Budak et al. (2021) reported a range between 11.8% and 14.4% for kefir‐based quark cheese. The protein content of white cheese was given as 13.93%–15.34% by Ektik (2022). The different protein ratio of the milk used in production and production methods leads to different protein ratios in both kefir and white cheese. Milk quality and safety are important for the revitalization of the dairy industry (Xiong, Chen, et al. 2022; Xiong, Wen, et al. 2022).

The highest ash value was observed in K0 (2.83%), and the lowest in K100 (1.48%) (Table 2). As the amount of concentrated kefir used increased, the ash value decreased (*p* < 0.05). Aktaş et al. (2024) reported the ash value of cow's milk kefir filtered using a cloth bag for 24 h at 4°C as 0.66%–0.73%, which is lower than our study. The removed water ratio, that is, dry matter, is effective in this. Indeed, Aktaş et al. (2024) reported the dry matter ratio of spreadable kefir as 12.58%. However, in our study, concentrated kefir has approximately 34% dry matter. Akan and Kinik (2018) reported ash values in white cheese as 2.52–3.36. Calcium chloride and salt (NaCl) used in the production of white cheese increased the ash values of the final product. The salt value, which was 0.86% in concentrated kefir, increased to 2.08% in white cheese, as also given in Table 2.

The use of concentrated kefir in cheese ball increased carbohydrate values (from 3.27 to 4.23) (Table 2). In cheese production, most of the lactose is separated with whey, and the remaining part in the cheese is converted into lactic acid by starter bacteria (Hayaloğlu and Özer 2011). In kefir, some of the lactose is used as a food source by microorganisms. As a result of fermentation of milk with kefir culture, the lactose ratio decreased from 4703 to 3314 mg/100 mL (Gamba et al. 2020). As a result, the cheese ball samples made from white cheese only had lower carbohydrate levels. The results are consistent with the 3.0%–3.2% value observed by Cebeci et al. (2020) in white cheese.

K0 was the group with the lowest energy value (194.44 kcal), whereas K100 had the highest energy value (209.45 kcal) (Table 2). The cheese balls' energy value increased with the percentage of concentrated kefir used. Carbohydrates, fats, and proteins are effective in the total calories of cheese balls. As the fat values of cheese ball samples are between 15% and 15.50%, fat is not responsible for the difference in energy values. The increase in protein and carbohydrate values from K0 to K100 increased the energy values.

Regarding the average pH values, the highest value is 4.50 (K75 and K100) and the lowest value is 4.40 (K0) (*p* < 0.05). pH is an important quality factor in cheese as it affects its textural, rheological, and melt properties (Ahsan et al. 2023). Demir and Özkısa (2020) reported the pH value range as 4.39–4.63 in concentrated kefir samples, and Aktaş et al. (2024) as 4.44–4.56 in spreadable kefir. Şanlı and Anlı (2020) found the pH of Çökelek cheese, which they produced using kefir, between 4.38 and 4.42. White cheese's pH ranged from 4.32 to 5.18, according to Akan and Kınık (2018). Our measured pH results are consistent with these studies.

The use of concentrated kefir in cheese ball reduced salt values (Table 2). The K100 had the lowest salt level (0.86%) and the K0 had the greatest salt content (2.08%) (*p* < 0.05). The reason for this is that white cheese has a high salt content and we add the least amount of salt to concentrated kefir within acceptable limits. In our study, we added 1% salt to concentrated kefir. This product had an acceptable salt concentration, according to the sensory analysis. As high salt causes hypertension and related health problems, excessive consumption should be avoided. Although sensitive to excessive sodium intake and salt content, sodium chloride serves several essential functions in cheese. Salt improves flavor and aroma profiles, regulates texture, final pH, water activity, and affects microbial growth and shelf life (Tidona et al. 2022). The salt content in white cheese was found to be 2.04%–3.23% by Çetinkaya (2021), and 1.85%–2.14% by Barac et al. (2016). These results are consistent with K0. Reducing the salt content in the cheese ball to an acceptable low level (from 2.08% to 0.86%) is an important result of our study.

Table 2 shows that the salt in dry matter content decreased from 6.42% in K0 to 2.47% in K100 (*p* < 0.05). As the concentrated kefir content in the cheese ball composition increased, the salt in dry matter of the cheese balls decreased (*p* < 0.05). With the increase in the use of concentrated kefir in cheese ball production, the increase in dry matter and the decrease in salt content were effective in obtaining this result. The results agree with findings from other white cheese studies (Çetinkaya 2021; Aydemir 2018).

### 3.2 Color Properties

Color analysis results of cheese ball samples are given in Table 3.

**TABLE 3:** Color properties of cheese balls.

	Cheese ball samples ($\bar{X}$ + SD)				
	K0	K25	K50	K75	K100
L*	95.53 ± 0.150^a^	95.63 ± 0.592^a^	95.78 ± 0.566^a^	95.76 ± 0.256^a^	95.74 ± 0.323^a^
a*	−2.30 ± 0.034^a^	−2.21 ± 0.093^a^	−1.79 ± 0.099^b^	−1.90 ± 0.039^b^	−1.88 ± 0.058^b^
b*	9.47 ± 0.311^a^	9.65 ± 0.238^a^	9.64 ± 0.198^a^	9.85 ± 0.053^ab^	10.27 ± 0.27^b^
C*	9.75 ± 0.308^a^	9.90 ± 0.252^a^	9.81 ± 0.215^a^	10.04 ± 0.059^ab^	10.45 ± 0.274^b^
WI	89.28 ± 0.256^a^	89.17 ± 0.449^a^	89.31 ± 0.033^a^	89.11 ± 0.086^a^	88.72 ± 0.368^a^
Δ*E*	—	0.49 ± 0.212^a^	0.85 ± 0.240^a^	0.65 ± 0.106^a^	0.98 ± 0.311^a^

*Note:* Means with different letters are statistically different (*p* < 0.05).

Abbreviations: $\bar{X}$: mean; SD, standard deviation.

Cheese color contributes significantly to sensory responses and is very important in the selection of food ingredients. As consumer expectations are affected by cheese color, measuring color characteristics is technologically and commercially important (Ricci et al. 2022). The color of cheese is affected by the type of milk used, the diet of the animal from which it is obtained, the fat content of the milk, the production method, and the colorants used. Colorants can be obtained from foods or produced from microbial sources (İncili et al. 2025). The *L* value consists of a scale ranging from 0 (black) to 100 (white). The L* values of the samples are between 95.53 and 95.78. The difference between the L* values of the cheese ball samples was statistically insignificant (*p* > 0.05). Aydemir and Kurt (2020) reported the L* value of white cheese as 93.47–94.38, whereas Gürel et al. (2021) 94.95 for kefir made from cow's milk. Factors, such as the type of milk used in production, the fat content of the milk, season, and feeding, significantly affect the color value in cheese. Aktaş et al. (2024) reported the L* values of spreadable kefir produced from cow's milk as 90.88–91.55, which is lower than our study. In this study, as the raw milk was heated at 90°C for 10 min, more nonenzymatic browning reactions occur and lead to a decrease in the L* value. However, we used UHT milk. The use of UHT milk is also advantageous in terms of food safety. For example, the concentration of aflatoxin M1 is significantly lower in UHT milk compared with pasteurized milk (Xiong, Chen, et al. 2022; Xiong, Wen, et al. 2022).

The a* value increased from −2.30 in K0 to −1.88 in K100 (*p* < 0.05). A positive a* value indicates redness, and a negative a* value indicates greenness (Giusti et al. 2024). It is seen that the a* value linearly approaches 0 (greenness decreases) when the concentrated kefir ratio used to make cheese balls increases (Table 3). Riboflavin gives a yellow‐green color. The riboflavin content may increase because of the activities of some lactic acid bacteria used in cheese production. This situation caused cheese balls made with only white cheese to have more greenness (Güzeler et al. 2017).

The use of concentrated kefir in cheese ball production increased the b* value (Table 3). The highest value was 10.27 in K100 and the lowest in K0 as 9.47 (*p* < 0.05). Positive *b* values indicate yellowness and negative *b* values indicate blueness. Milk carotenoids are responsible for the yellow color of cow's milk (Milovanovic et al. 2020). During the production of white cheese, the separation of some of the whey and color substances, such as carotene, riboflavin cause the b* value to be lower in white cheese. Our results were close to the value of 9.88–10.57 found by Aktaş et al. (2024) in spreadable kefir produced from cow's milk and the value of 9.09 found by Aydemir and Kurt (2020) in white cheese.

Chroma (C*) represents the vividness or saturation of a color (Milovanovic et al. 2020). As seen in Table 3, C* values were between 9.75 (K0) and 10.45 (K100). The C* values of cheese balls produced using concentrated kefir are higher than the control. The differences in the a* and b* values of the samples due to the reasons mentioned above have led to differences in the C* values. Aydemir and Kurt (2020) found the C* color value of white cheese as 7.38–9.11. Aktaş et al. (2024) reported the C* value as 10.46–11.12 in spreadable kefir made from cow's milk. Our results are consistent with these studies.

The whiteness index (WI) is used to indicate the degree of whiteness, which is the critical color characteristic of dairy products, and mathematically combines lightness and yellow‐blue into a single term (Milovanovic et al. 2020). The WI value of the cheese ball samples was between 88.72 (K100) and 89.31 (K50). The difference between the WI values of the samples is insignificant (*p* > 0.05). WI below 100 indicates yellowish white color; above 100 represents bluish white color (Ramírez‐Rivas et al. 2022). Aktaş et al. (2024) reported the WI value in spreadable kefir made from cow's milk as 85.68–86.51, which is close to our study.

Regarding Δ*E* results calculated to measure the color difference between the control group K0 and the other groups, it is seen that the ΔE value increases with the increase in the concentrated kefir usage rate. However, this change is statistically insignificant (*p* > 0.05). Differences in color are considered very significant if Δ*E* > 3, significant if 1.5 < Δ*E* < 3, and minor if 1.5 < Δ*E* (Zhang et al. 2023). The ΔE values observed in our cheese ball samples were less than 1.5, indicating that there was a small color difference between the control and the other groups.

### 3.3 Textural Properties

Hardness, adhesiveness, springiness, cohesiveness, gumminess, and chewiness values of cheese ball samples are given in Table 4.

**TABLE 4:** Textural properties of cheese ball samples.

	Cheese ball samples ($\bar{X}$ + SD)				
	K0 (Control)	K25	K50	K75	K100
Hardness (g)	115.17 ± 11.061^ab^	67.38 ± 6.35^c^	89.58 ± 2.391^a^	132.83 ± 8.056^bd^	219.36 ± 31.666^d^
Adhesiveness (g.s)	−171.65 ± 15.175^d^	−276.59 ± 26.334^c^	−373.93 ± 23.366^ab^	−523.25 ± 68.431^a^	−293.37 ± 54.364^bcd^
Springiness (s)	0.38 ± 0.031^a^	0.85 ± 0.014^c^	0.89 ± 0.024^c^	0.90 ± 0.006^c^	0.45 ± 0.021^b^
Cohesiveness	0.32 ± 0.01^a^	0.64 ± 0.025^c^	0.64 ± 0.056^c^	0.63 ± 0.041^c^	0.42 ± 0.023^b^
Gumminess (g)	36.30 ± 2.584^a^	42.93 ± 5.347^ab^	57.54 ± 5.667^bc^	83.06 ± 8.432^d^	91.47 ± 15.043^cd^
Chewiness (g.s)	13.77 ± 1.305^a^	36.47 ± 5.168^b^	51.25 ± 4.593^c^	74.47 ± 7.884^d^	41.52 ± 7.342^bc^

*Note:* Means with different letters are statistically different (*p* < 0.05).

Abbreviations: $\bar{X}$: mean; SD, standard deviation.

Regarding the hardness values, the highest value was for the K100 (219.36 g) and the lowest for the K25 (67.38 g). As the concentrated kefir ratio increased, the hardness value increased. We attribute this to the increase in dry matter and protein values with the increase in concentrated kefir ratio. Zheng et al. (2016) reported a negative correlation between moisture content and firmness values of cheeses. The hardness value increased as a result of the effect of the increase in protein ratio on water retention capacity. Dabour and El‐Shanshory (2018) reported that hardness values increased significantly with increasing protein content in spreadable processed cheese (*p* < 0.05). In cheese, the network created by the protein holds other milk components. While high protein and pH increase hardness, other components (water, fat and salt) decrease hardness (Hayaloğlu and Özer 2011). As seen in Table 2, the high protein and pH values of K100, on the contrary, the high water and salt content of K0 caused K100 to have a higher hardness value. Demir and Özkısa (2020) reported the hardness value of the filtered kefir as 165.78 g. In this study, the straining process up to 20% dry matter resulted in lower hardness values than in our study.

**FIGURE 1:**
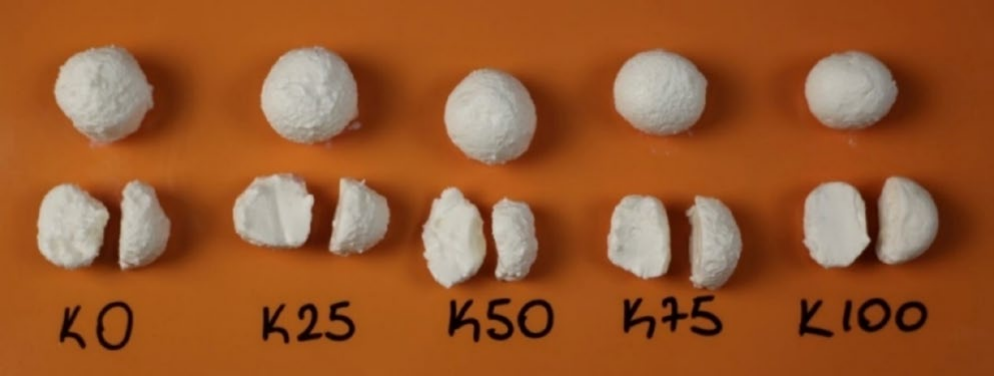
Cheese ball samples.

It was clear from the data in Table 4 cheese balls produced with concentrated kefir had higher adhesiveness, springiness, cohesiveness, and chewiness values than the control (white cheese only) (*p* < 0.05). The highest gumminess value was for K100 (91.47 g) and the lowest for K0 (36.30 g) (*p* < 0.05). The gumminess value of cheese balls increased by using concentrated kefir. The main reason for the difference in textural parameters is that the protein and dry matter ratios of cheese ball samples produced using concentrated kefir are higher than those of the control. Protein and fat have a great effect on cheese texture. As seen in Table 2, as there is no significant difference between the fat valuesof cheese ball samples, the differences in the textural properties of cheese ball samples are largely due to the protein ratio. Dabour and El‐Shanshory (2018) reported that cohesiveness, gumminess, and chewiness values increased significantly with the increase in protein ratio in spreadable processed cheese (*p* < 0.05). Filtering kefir with a centrifuge endows it with an adhesive character (Figure 1). We realized this increased adhesiveness while shaping the samples by hand and during sensory analysis as well. Aydemir and Kurt (2020) determined the springiness and cohesiveness of white cheese as 0.83–0.87 s and 0.71–0.75, respectively. Akan and Kinik (2018) reported the chewiness value of white cheese as 11.85–37.46.

### 3.4 Rheological Properties

**FIGURE 2:**
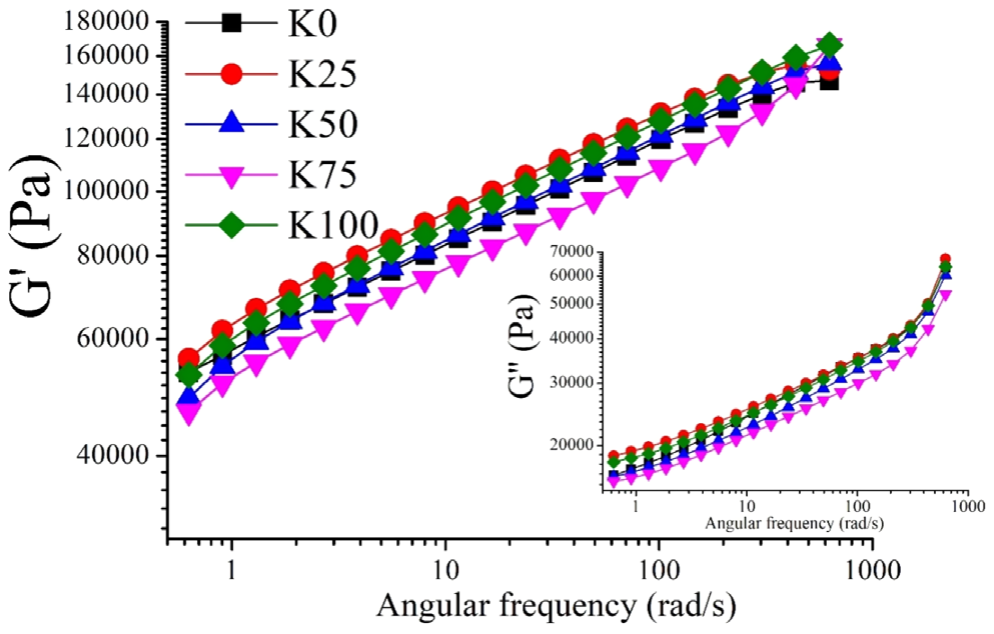
Storage modulus (*G*′) and Loss modulus (*G*″) values of cheese balls.

The rheological analysis results are given in Figure 2. For all the cheese ball samples, the Storage modulus (*G*′) and Loss modulus (*G*″) values increased with increasing angular frequency. The *G*′ value was higher than the *G*″ value at every frequency, and there was no phase transition between *G*′ and *G*″, indicating that the structure exhibited a viscoelastic solid character. A material is more solid if its *G*′ value is higher, and more liquid if its *G*″ value is higher. As can be seen from Figure 2, *G*′ was more influenced by the frequency increase compared with *G*″. K75 was the lowest *G*′ and *G*″ values, indicating that K75 had less viscoelasticity than the other samples. Additionally, K25 and K100 had the highest *G*′ values. The high amount of protein and ash and the low amount of fat in fresh cheese lead to the result of *G*′ > *G*″ (Joshi et al. 2004) which, in turn, indicates a strong cheese texture and flexibility (Sheikh, Hasan, et al. 2023; Sheikh, Hasani, et al. 2023). Also, the tan*δ* < 1 in all samples shows that the samples have viscoelastic character. Similar viscoelastic behavior was reported in white cheese by Aydemir and Kurt (2020) and Sheikh, Hasan, et al. (2023) and Sheikh, Hasani, et al. (2023). Cheese's viscoelastic behavior is influenced by its protein and fat content as well as by its moisture content (Dimitreli and Thomareis 2008). The protein network structure is responsible for the viscoelastic character of cheese.

**FIGURE 3:**
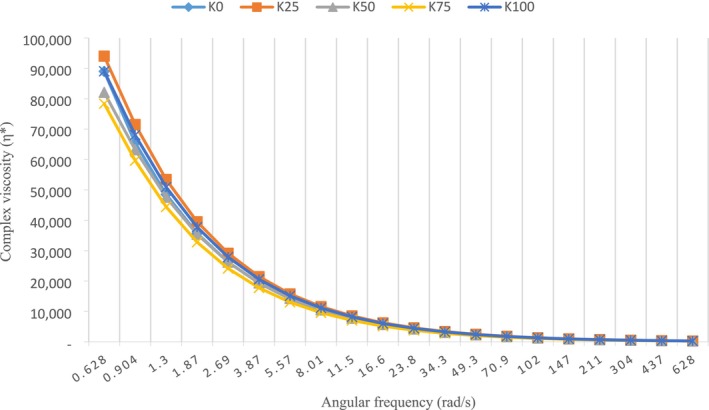
Complex viscosity (η*) values of cheese balls.

Complex viscosity (η*) is a measure of the hardness of the material. η* decreases with increasing frequency, indicating non‐Newtonian behavior (Sheikh, Hasan, et al. 2023; Sheikh, Hasani, et al. 2023). K25 and K100 were the samples with the highest η* values (Figure 3). The high protein and dry matter values of K100 caused it to absorb more water and therefore to have a higher complex viscosity. Dimitreli and Thomareis (2008) reported that cheeses with low moisture content showed higher complex viscosity values than cheeses with high moisture content.

### 3.5 Sensory Properties

**FIGURE 4:**
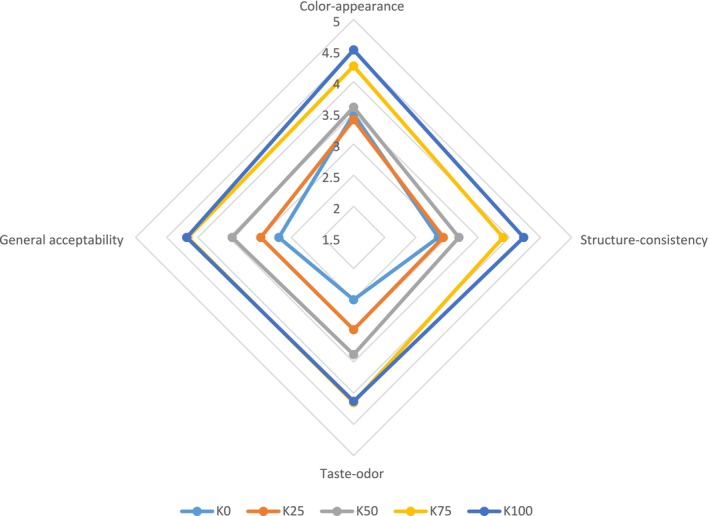
Sensory analysis results of cheese ball samples.

Sensory analysis results of cheese ball samples are presented in Figure 4.

The highest color‐appearance value was for the K100 (4.51), whereas the lowest was for the K0 (3.48) (*p* < 0.05). It is seen from the data that the color‐appearance values increased as the amount of concentrated kefir used in cheese ball production increased. Figure 1 shows that the smoother surface of the K100 group yields higher color‐appearance scores. This is thought to be due to the concentration of kefir by centrifugation. The fact that the L* value was the highest in K100 in the color analysis supports the finding that K100 received higher color‐appearance scores in the sensory evaluation (Table 3). Similarly, Öztürkoğlu‐Budak et al. (2021) determined that Quark cheeses produced from kefir and yayık buttermilk were more appreciated than cheeses made from skim milk in terms of color‐appearance.

The use of concentrated kefir in cheese ball production increased the structure‐consistency scores. The structure‐consistency score, which was 2.86 in K0, increased to 4.23 in K100 with a linear increase (*p* < 0.05). Proteins positively affect the structure‐consistency because of their water retention properties. The fact that K100 was the group with the highest protein and dry matter values and therefore the highest hardness value was effective in the high structure‐consistency scores (Tables 2 and 4). Consistent with our study, Öztürkoğlu‐Budak et al. (2021) found Quark cheeses prepared from kefir and yayık buttermilk to be more preferable in terms of body‐texture than cheeses prepared from skim milk.

Taste‐odor data showed that K75 was the most liked group, followed by K100 (*p* > 0.05). The use of concentrated kefir in cheese ball production caused a statistically significant increase in taste‐odor scores (*p* < 0.05). The differences in taste and aroma caused by some metabolites produced by lactic acid bacteria and yeasts in the composition of the starter culture used in kefir production have led to a higher appreciation of cheese ball samples produced using kefir. Lactic, succinic, pyruvic, ketoglutaric, and oxalic acids formed during fermentation provide a refreshing and acidic taste of kefir. Typical kefir aroma is affected by a significant amount of ethanol and carbon dioxide produced by yeasts during fermentation (Saygili et al. 2023). Lactic acid gives a sour taste to kefir (Rutkowska et al. 2022). The difference in taste between cheese ball samples is also due to the different methods of coagulation of white cheese and kefir used in production. White cheese is enzyme‐coagulated, whereas kefir is acid‐coagulated. Demirtaş and Coşkun (2018) reported that acid‐coagulated tulum cheeses were more appreciated in terms of taste than those that were enzyme‐coagulated. In a study, it was reported that the taste and odor scores increased with the use of kefir in çökelek cheese on the 14th and 28th days of storage (Şanlı and Anlı 2020). Öztürkoğlu‐Budak et al. (2021) found that Quark cheeses produced from kefir and yayık buttermilk had better odor‐flavor‐taste than cheese manufactured from skim milk.

In cheese ball samples, the highest general acceptability value was for K100 (4.18), whereas the lowest was for K0 (2.70) (*p* < 0.05). The use of concentrated kefir in cheese ball production increased the general acceptability scores. The increase in panelist scores in color‐appearance, structure‐consistency, and taste‐odor parameters with the use of concentrated kefir also led to an increase in general acceptability scores. We think that the coagulation method of white cheese and kefir is also effective in this. Demirtaş and Coşkun (2018) reported that the acid‐coagulated cheese sample was the most preferred cheese in terms of general appreciation. Şanlı and Anlı (2020) reported that the cheese produced from kefir was more appreciated in terms of general acceptability than the one produced from yogurt.

## 4 Conclusion

In this study, we produced cheese balls by combining concentrated kefir and white cheese in various ratios (0:100, 25:75, 50:50, 75:25, and 100:0) to improve the consumption diversity of kefir. Physicochemical, textural, rheological, and sensory properties of cheese ball samples were examined. Our results showed that K100 had the highest dry matter, protein, energy, pH, b*, C*, Δ*E*, hardness, and gumminess values. K25 and K100 were the samples with the highest *G*′ and η* values. As a result of sensory analyses, the highest scores in terms of color‐appearance, structure‐consistency, and general acceptability criteria were for K100 and K75, respectively. We determined that dry matter, protein, carbohydrate, energy, pH, springiness, cohesiveness, gumminess, chewiness, and sensory appreciation values increased, whereas fat in dry matter, ash, salt, and salt in dry matter content decreased as a result of the use of concentrated kefir in cheese ball production. A small color difference (Δ*E* < 1.5) was detected between the samples using concentrated kefir and the control group. Reducing the salt content in the cheese ball to an acceptable low level (from 2.08% at K0 to 0.86% at K100) is an important result of our study. Concentrated kefir can be used alone or mixed with white cheese in different proportions to obtain cheese balls with high sensory, nutritional, and functional properties. Centrifuge parameters can be changed to obtain higher or lower dry matter values for centrifuged kefir and cheese.

## Author Contributions

**Hilal Çavuş:** conceptualization (supporting), investigation (equal), resources (equal), writing – original draft (equal). **Ahmet Emirmustafaoğlu:** data curation (supporting), formal analysis (supporting), methodology (lead), project administration (lead), supervision (supporting), validation (supporting), writing – review and editing (supporting).

## Ethics Statement

The study was approved by the Bolu Abant İzzet Baysal University Ethics Committee with registration number: 2022/41.

## Conflicts of Interest

The authors declare no conflicts of interest.

## Data Availability

The data that support the findings of this study are available upon request from the corresponding author.

## References

[fsn370183-bib-0001] Abedinia, A. , R. A. Zambelli , and E. Hosseini . 2025. “Functional Foods for Oral and Dental Health.” In The Power of Functional Foods and Novel Bioactives, edited by Unleashing , 337–353. Academic Press. 10.1016/B978-0-443-28862-3.00017-0.

[fsn370183-bib-0002] Ahsan, M. , T. M. Ali , and A. Hasnain . 2023. “Use of Oxidized Potato Starch as Simultaneous Fat and Casein Replacer in Analogue Mozzarella Cheese‐II: Impact on Functional and Sensory Properties of Cheese.” Food Hydrocolloids 142: 108810. 10.1016/j.foodhyd.2023.108810.

[fsn370183-bib-0003] Akan, E. , and O. Kinik . 2018. “Effect of Mineral Salt Replacement on Properties of Turkish White Cheese.” Mljekarstvo 68, no. 1: 46–56. 10.15567/mljekarstvo.2018.0106.

[fsn370183-bib-0004] Aktaş, H. , H. M. Aktaş , B. Ürkek , M. Şengül , and B. Çetin . 2024. “Evaluation of Spreadable Kefir Produced From Different Milks in Terms of Some Quality Criteria.” Probiotics and Antimicrobial Proteins 16, no. 5: 1–10. 10.1007/s12602-023-10129-8. 37523112

[fsn370183-bib-0005] Amador‐Espejo, G. G. , I. I. Ruiz‐Lopez , P. J. Gibbens‐Bandala , R. J. Delgado‐Macuil , and H. Ruiz‐Espinosa . 2021. “Thermosonicated Whey Protein Concentrate Blends on Quality Attributes of Reduced Fat Panela Cheese.” Ultrasonics Sonochemistry 76: 105621. 10.1016/j.ultsonch.2021.105621. 34144445 PMC8217677

[fsn370183-bib-0006] Aydemir, O. 2018. “Proteolysis and Lipolysis of White‐Brined (Beyaz) Cheese During Storage: Effect of Milk Pasteurization Temperature.” Journal of Food Processing and Preservation 42, no. 5: e13612. 10.1111/jfpp.13612.

[fsn370183-bib-0007] Aydemir, O. , and A. Kurt . 2020. “The Effect of Different Pasteurization Conditions on the Rheological, Textural and Sensory Properties of White Cheese.” The Journal of Food 45, no. 6 1083–1096. 10.15237/gida.GD20101.

[fsn370183-bib-0008] Azizi, N. F. , M. R. Kumar , S. K. Yeap , et al. 2021. “Kefir and Its Biological Activities.” Food 10, no. 6: 1210. 10.3390/foods10061210. PMC8226494 34071977

[fsn370183-bib-0009] Barac, M. , M. Pesic , S. Zilic , et al. 2016. “Protein Profiles and Total Antioxidant Capacity of Water‐Soluble and Water‐Insoluble Fractions of White Brined Goat Cheese at Different Stages of Ripening.” International Journal of Food Science & Technology 51, no. 5: 1140–1149. 10.1111/ijfs.13091.

[fsn370183-bib-0010] Cebeci, A. , M. Yaman , B. Yalcin , and F. E. Gunes . 2020. “Determination of Carbohydrate Amounts of Various Cheese Types Presented to Sale in the Market.” International Journal of Food Sciences and Nutrition 5, no. 6: 30–35.

[fsn370183-bib-0011] Černíková, M. , J. Nebesářová , R. N. Salek , L. Řiháčková , and F. Buňka . 2017. “Microstructure and Textural and Viscoelastic Properties of Model Processed Cheese With Different Dry Matter and Fat in Dry Matter Content.” Journal of Dairy Science 100, no. 6: 4300–4307. 10.3168/jds.2016-12120. 28390721

[fsn370183-bib-0012] Çetinkaya, A. 2021. “Determination of Physical and Chemical Properties of Yoghurts, White Cheese and Kars Kashar Cheese Sold in Kars, Turkey.” The Journal of Food 46, no. 5: 1233–1242. 10.15237/gida.GD21060.

[fsn370183-bib-0013] Chudy, S. , A. Makowska , M. P. Tek , and M. K. N. Bartkowiak . 2019. “Application of Microwave Vacuum Drying for Snack Production: Characteristics of Pure Cheese Puffs.” International Journal of Dairy Technology 72, no. 1: 82–88. 10.1111/1471-0307.12562.

[fsn370183-bib-0014] Dabour, N. , and A. El‐Shanshory . 2018. “The Influence of Protein Content and Some Hydrocolloids on Textural Attributes of Spreadable Processed Cheese.” Applied Science Research 14: 5–11. 10.22587/jasr.2018.14.5.2.

[fsn370183-bib-0015] Demir, M. , and D. Özkısa . 2020. “Determination of Some Physicochemical and Microbiological Properties of Kefir Concentrated by Different Methods.” Mediterranean Agricultural Sciences 33, no. 2: 239–246. 10.29136/mediterranean.697454.

[fsn370183-bib-0016] Demirtaş, M. , and H. Coşkun . 2018. “The Changes During Ripening of Tulum Cheeses Produced by Different Coagulation Methods From Goat Milk.” The Journal of Food 43, no. 5: 835–845. 10.15237/Gida.GD18073.

[fsn370183-bib-0017] Dimitreli, G. , and A. S. Thomareis . 2008. “Effect of Chemical Composition on the Linear Viscoelastic Properties of Spreadable‐Type Processed Cheese.” Journal of Food Engineering 84, no. 3: 368–374. 10.1016/j.jfoodeng.2007.05.030.

[fsn370183-bib-0018] Ektik, N. 2022. “Isolation and Identification of Lactic Acid Bacteria from Different Stages of Classic (Ripened) White Cheese Production and Determination of Technological Characteristics,” Balikesir University (Turkey) ProQuest Dissertations & Theses.

[fsn370183-bib-0019] Emirmustafaoğlu, A. , and H. Coşkun . 2017. “Optimization of Production Technology of keş for Frying.” Yuzuncu Yil University Journal of Agricultural Sciences 27, no. 3: 357–369. 10.29133/yyutbd.291927.

[fsn370183-bib-0020] Farag, M. A. , S. A. Jomaa , A. Abd El‐Wahed , and A. H. R. El‐Seedi . 2020. “The Many Faces of Kefir Fermented Dairy Products: Quality Characteristics, Flavour Chemistry, Nutritional Value, Health Benefits, and Safety.” Nutrients 12, no. 2: 346. 10.3390/nu12020346. 32013044 PMC7071183

[fsn370183-bib-0021] Gamba, R. R. , S. Yamamoto , M. Abdel‐Hamid , et al. 2020. “Chemical, Microbiological, and Functional Characterization of Kefir Produced From Cow's Milk and Soy Milk.” International Journal of Microbiology 2020, no. 1: 7019286. 10.1155/2020/7019286. 32565815 PMC7269609

[fsn370183-bib-0022] Ganatsios, V. , P. Nigam , S. Plessas , and A. Terpou . 2021. “Kefir as a Functional Beverage Gaining Momentum Towards Its Health Promoting Attributes.” Beverages 7, no. 3: 48. 10.3390/beverages7030048.

[fsn370183-bib-0023] García‐Burgos, M. , J. Moreno‐Fernández , M. J. Alférez , J. Díaz‐Castro , and I. López‐Aliaga . 2020. “New Perspectives in Fermented Dairy Products and Their Health Relevance.” Journal of Functional Foods 72: 104059. 10.1016/j.jff.2020.104059.

[fsn370183-bib-0024] García‐Gómez, B. , L. Vázquez‐Odériz , N. Muñoz‐Ferreiro , Á. Romero‐Rodríguez , and M. Vázquez . 2020. “Rennet Type and Microbial Transglutaminase in Cheese: Effect on Sensory Properties.” European Food Research and Technology 246: 513–526. 10.1007/s00217-019-03418-6.

[fsn370183-bib-0025] Giusti, M. M. , B. Gordillo , and M. L. González‐Miret . 2024. “Color Analysis.” In Nielsen's Food Analysis, 509–522. Springer International Publishing.

[fsn370183-bib-0026] Gümüş, P. , and A. A. Hayaloğlu . 2019. “Effects of Blends of Camel and Calf Chymosin on Proteolysis, Residual Coagulant Activity, Microstructure, and Sensory Characteristics of White Cheese.” Journal of Dairy Science 102, no. 7: 5945–5956. 10.3168/jds.2018-15671. 31079909

[fsn370183-bib-0027] Gürel, D. B. , M. Ildız , S. Sabancı , N. Koca , Ö. Çağındı , and F. İçier . 2021. “The Effect of Using Cow and Goat Milk on Antioxidant, Rheological and Sensory Properties of Kefir.” Turkish Journal of Agriculture‐Food Science and Technology 9, no. 1: 7–14. 10.24925/turjaf.v9i1.7-14.3330.

[fsn370183-bib-0028] Güzeler, N. , E. M. Esmek , and M. Kalender . 2017. “Peyniraltı suyu ve peyniraltı suyunun içecek sektöründe değerlendirilme olanakları.” Çukurova Tarım Ve Gıda Bilimleri Dergisi 32, no. 2: 27–36.

[fsn370183-bib-0029] Hayaloğlu, A. A. , and B. Özer . 2011. Fundamentals of Cheese Science, 643. Sidas Media Ltd (Chapter 1).

[fsn370183-bib-0030] İncili, G. K. , R. Razavi , A. A. Hayaloğlu , A. Abedinia , S. S. Mirmoeini , and M. Moradi . 2025. “Microbial Derived Biomaterials: Fabrication, Processing, and Food Application.” In Sustainable Materials for Food Packaging and Preservation, 55–84. Elsevier. 10.1016/B978-0-443-13567-5.00003-4.

[fsn370183-bib-0031] Joshi, S. , B. Davis , M. Jomier , and G. Gerig . 2004. “Unbiased Diffeomorphic Atlas Construction for Computational Anatomy.” NeuroImage 23: 151–160. 10.1016/j.neuroimage.2004.07.068. 15501084

[fsn370183-bib-0032] La Torre, C. , P. Caputo , E. Cione , and A. Fazio . 2024. “Comparing Nutritional Values and Bioactivity of Kefir From Different Types of Animal Milk.” Molecules 29, no. 11: 2710. 10.3390/molecules29112710. 38893583 PMC11173642

[fsn370183-bib-0033] Lakshmi, T. S. , A. MaryPramela , and P. Iyer . 2017. “Anti‐Microbial, Anti‐Fungal and Anti‐Carcinogenic Properties of Coconut Milk Kefir.” International Journal of Home Science 3: 365–369.

[fsn370183-bib-0034] Li, T. , Q. Guo , M. Liang , Y. Qu , Y. Zhang , and Q. Wang . 2023. “Impact of Additives on the Formation of Thermally Induced Trans Linoleic Acid in Peanut Oil.” International Journal of Food Science and Technology 58, no. 5: 2498–2504. 10.1111/ijfs.16391.

[fsn370183-bib-0035] Li, Z. , F. Xiang , X. Huang , et al. 2024. “Properties and Characterization of Sunflower Seeds From Different Varieties of Edible and Oil Sunflower Seeds.” Food 13, no. 8: 1188. 10.3390/foods13081188. PMC11048903 38672861

[fsn370183-bib-0036] Lorenzen, M. , A. Tică , S. K. Lillevang , E. J. Windhab , and L. Ahrné . 2025. “The Influence of Milk Fat Content on the Extrusion of Rennet Casein Emulsion Gels.” Food Hydrocolloids 163: 111109. 10.1016/j.foodhyd.2025.111109.

[fsn370183-bib-0037] Mazinani, S. , A. Motamedzadegan , S. Nghizadeh Raeisi , and M. Alimi . 2021. “Characterization of Bacteriologically Acidified Feta Cheese Using Soy Protein Isolate in Different Substitution Percentages: Rheological, Microbiological and Sensory Properties.” Journal of Food Measurement and Characterization 15, no. 6: 5515–5527. 10.1007/s11694-021-00973-z.

[fsn370183-bib-0038] Metin, M. , and G. F. Öztürk . 2017. Süt ve mamülleri analiz yöntemleri. Ege University Faculty of Engineering Publications.

[fsn370183-bib-0039] Milovanovic, B. , I. Djekic , J. Miocinovic , et al. 2020. “What Is the Color of Milk and Dairy Products and How Is It Measured?” Food 9, no. 11: 1629. 10.3390/foods9111629. PMC7695135 33171601

[fsn370183-bib-0040] Mitra, S. , and B. C. Ghosh . 2021. “Kefir–a Fermented Milk Product Beneficial for Gastrointestinal Health.” Indian Journal of Dairy Science 74, no. 6: 469–478. 10.33785/IJDS.2021.v74i06.001.

[fsn370183-bib-0041] Nasiri, E. , J. Hesari , S. S. Shekarforoush , S. Azadmard Damirchi , S. Gensberger‐Reigl , and M. Pischetsrieder . 2020. “Novel Milk‐Clotting Enzyme From Sour Orange Flowers ( *Citrus aurantium* L.) as a Coagulant in Iranian White Cheese.” European Food Research and Technology 246: 139–148. 10.1007/s00217-019-03403-z.

[fsn370183-bib-0042] Ozer, B. H. 2006. Yogurt Science and Technology. Sidas Media Ltd.

[fsn370183-bib-0043] Öztürkoğlu‐Budak, S. , H. C. Akal , and N. Türkmen . 2021. “Use of Kefir and Buttermilk to Produce an Innovative Quark Cheese.” Journal of Food Science and Technology 58, no. 1: 74–84. 10.1007/s13197-020-04516-0. 33505053 PMC7813949

[fsn370183-bib-0044] Qi, W. , T. Li , Z. Zhang , and T. Wu . 2021. “Preparation and Characterization of Oleogel‐In‐Water Pickering Emulsions Stabilized by Cellulose Nanocrystals.” Food Hydrocolloids 110: 106206. 10.1016/j.foodhyd.2020.106206.

[fsn370183-bib-0045] Rajoka, M. S. R. , H. M. Mehwish , H. Fang , et al. 2019. “Characterization and Anti‐Tumoractivity of Exopoly Saccharide Produced by *Lactobacillus kefiri* Isolated From Chinese Kefir Grains.” Journal of Functional Foods 63: 103588. 10.1016/j.jff.2019.103588.

[fsn370183-bib-0046] Ramírez‐Rivas, I. K. , N. Gutiérrez‐Méndez , A. L. Rentería‐Monterrubio , et al. 2022. “Effect of Different Types and Concentrations of Salts Added to Requeson Cheese on Texture, Sensory, and Physiochemical Characteristics.” Journal of Food Processing and Preservation 46, no. 4: e16336. 10.1111/jfpp.16336.

[fsn370183-bib-0047] Ricci, M. , F. Gasperi , I. Endrizzi , et al. 2022. “Effect of Dairy, Season, and Sampling Position on Physical Properties of Trentingrana Cheese: Application of an LMM‐ASCA Model.” Food 11, no. 1: 127. 10.3390/foods11010127. PMC8750008 35010253

[fsn370183-bib-0048] Rutkowska, J. , A. Antoniewska‐Krzeska , A. Żbikowska , P. Cazón , and M. Vázquez . 2022. “Volatile Composition and Sensory Profile of Lactose‐Free Kefir, and Its Acceptability by Elderly Consumers.” Molecules 27, no. 17: 5386. 10.3390/molecules27175386. 36080153 PMC9457958

[fsn370183-bib-0049] Salari, A. , S. Ghodrat , A. Gheflati , L. Jarahi , M. Hashemi , and A. Afshari . 2021. “Effect of Kefir Beverage Consumption on Glycemic Control: A Systematic Reviewand Meta‐Analysis of Randomized Controlled Clinical Trials.” Complementary Therapies in Clinical Practice 44: 101443. 10.1016/j.ctcp.2021.101443. 34280689

[fsn370183-bib-0050] Şanlı, T. , and E. A. Anlı . 2020. “Use of Kefir in çokelek Cheese Production.” The Journal of Food 45, no. 1: 139–149. 10.15237/gida.GD19162.

[fsn370183-bib-0051] Saygili, D. , O. Yerlikaya , and A. Akpinar . 2023. “The Effect of Using Different Yeast Species on the Composition of Carbohydrates and Volatile Aroma Compounds in Kefir Drinks.” Food Bioscience 54: 102867. 10.1016/j.fbio.2023.102867.

[fsn370183-bib-0052] Sheikh, F. , M. Hasani , H. Kiani , M. JavadAsadollahzadeh , and J. Seyfi . 2023. “Investigation of Textural, Rheological and Sensory Properties of White Cheese Analog Containing Sesame Seeds Oleosome.” Journal of Food Measurement and Characterization 17, no. 1: 63–74. 10.1007/s11694-022-01582-0.

[fsn370183-bib-0053] Sheikh, M. C. , M. M. Hasan , M. N. Hasan , et al. 2023. “Toxic Cadmium (II) Monitoring and Removal From Aqueous Solution Using Ligand‐Based Facial Composite Adsorbent.” Journal of Molecular Liquids 389: 122854. 10.1016/j.molliq.2023.122854.

[fsn370183-bib-0054] Stone, H. , R. N. Bleibaum , and H. A. Thomas . 2020. Sensory Evaluation Practices. Academic Press.

[fsn370183-bib-0055] Tamime, A. Y. , R. K. Robinson , and M. Michel . 2007. Microstructure of Concentrated and Dried Milk Products, 104–133. Blackwell Publishing. 10.1016/j.foodchem.2014.11.017.

[fsn370183-bib-0056] Tidona, F. , M. Zago , D. Carminati , and G. Giraffa . 2022. “The Reduction of Salt in Different Cheese Categories: Recent Advances and Future Challenges.” Frontiers in Nutrition 9: 859694. 10.3389/fnut.2022.859694. 35445068 PMC9013816

[fsn370183-bib-0057] Turan, F. , and O. Türkay . 2024. “Food Waste Management in Hotels: Trabzon Example. Manas Journal of.” Social Research 13, no. 2: 700–717. 10.33206/mjss.1306637.

[fsn370183-bib-0058] Vieira, C. P. , A. I. L. Rosario , C. A. Lelis , et al. 2021. “Bioactive Compounds From Kefir and Their Potential Benefits on Health: A Systematic Review and Meta‐Analysis.” Oxidative Medicine and Cellular Longevity 2021: 1–34. 10.1155/2021/9081738. PMC8566050 34745425

[fsn370183-bib-0059] Viogenta, P. , N. Kartinah , A. Khairunnisa , and F. Rahman . 2021. “Quality of Peanut (Arachis Hypogeae L.) Kefir With Variation in Ragi Starter Concentrationand Long Fermentation.” Jurnal Biota 7, no. 2: 85–93. 10.19109/Biota.v7i2.8147.

[fsn370183-bib-0060] Xiong, J. , F. Chen , J. Zhang , et al. 2022. “Occurrence of Aflatoxin M1 in Three Types of Milk From Xinjiang, China, and the Risk of Exposure for Milk Consumers in Different Age‐Sex Groups.” Food 11, no. 23: 3922. 10.3390/foods11233922. PMC9738243 36496730

[fsn370183-bib-0061] Xiong, J. , D. Wen , H. Zhou , et al. 2022. “Occurrence of Aflatoxin M1 in Yogurt and Milk in Central‐Eastern China and the Risk of Exposure in Milk Consumers.” Food Control 137: 108928. 10.1016/j.foodcont.2022.108928.

[fsn370183-bib-0062] Yegin, Z. , M. N. Z. Yurt , B. B. Tasbasi , et al. 2022. “Determination of Bacterial Community Structure of Turkish Kefir Beverages via Metagenomic Approach.” International Dairy Journal 129: 105337. 10.1016/j.idairyj.2022.105337.

[fsn370183-bib-0063] Zhang, Y. , L. Wang , Y. Bu , et al. 2023. “Effects of Radio Frequency Heating on the Glass Transition, Protein Structure, and Volatile Compounds Profile of Commercial Powdered Infant Formula Milk.” Food Control 154: 109987. 10.1016/j.foodcont.2023.109987.

[fsn370183-bib-0064] Zheng, Y. , Z. Liu , and B. Mo . 2016. “Texture Profile Analysis of Sliced Cheese in Relation to Chemical Composition and Storage Temperature.” Journal of Chemistry 2016, no. 1: 8690380. 10.1155/2016/8690380.

